# Supplementary material for: Multi‐dataset identification of innovative feature genes and molecular mechanisms in keratoconus
Source: J Cell Mol Med. 2024 Sep 19;28(18):e70079. doi: 10.1111/jcmm.70079 (PMC11412914; doi:10.1111/jcmm.70079)
Supplement: Supplementary file 2 — Material S2. [file JCMM-28-e70079-s002.zip › 7.GSEA analysis/4.MYRF/KEGG_FC_EPSILON_RI_SIGNALING_PATHWAY.html]

Details for gene set KEGG\_FC\_EPSILON\_RI\_SIGNALING\_PATHWAY[GSEA]

|  || Dataset | MYRF.group.cls#h\_versus\_l |
| Phenotype | group.cls#h\_versus\_l |
| Upregulated in class | l |
| GeneSet | KEGG\_FC\_EPSILON\_RI\_SIGNALING\_PATHWAY |
| Enrichment Score (ES) | -0.42943665 |
| Normalized Enrichment Score (NES) | -1.4952006 |
| Nominal p-value | 0.022 |
| FDR q-value | 0.20656198 |
| FWER p-Value | 0.767 |
Table: GSEA Results Summary

  

Fig 1: Enrichment plot: KEGG\_FC\_EPSILON\_RI\_SIGNALING\_PATHWAY      
 Profile of the Running ES Score & Positions of GeneSet Members on the Rank Ordered List

  

| SYMBOL | TITLE | RANK IN GENE LIST | RANK METRIC SCORE | RUNNING ES | CORE ENRICHMENT || 1 | PLA2G4E | na | 485 | 0.577 | 0.0321 | No |
| 2 | PIK3R5 | na | 661 | 0.528 | 0.0693 | No |
| 3 | RAC3 | na | 2468 | 0.325 | 0.0431 | No |
| 4 | IL3 | na | 5237 | 0.215 | -0.0196 | No |
| 5 | INPP5D | na | 6653 | 0.174 | -0.0466 | No |
| 6 | SOS1 | na | 6706 | 0.172 | -0.0343 | No |
| 7 | PIK3R1 | na | 7105 | 0.161 | -0.0329 | No |
| 8 | IL13 | na | 7183 | 0.159 | -0.0224 | No |
| 9 | PLA2G3 | na | 8083 | 0.136 | -0.0375 | No |
| 10 | MAPK9 | na | 8409 | 0.129 | -0.0365 | No |
| 11 | MAPK12 | na | 8572 | 0.125 | -0.0312 | No |
| 12 | KRAS | na | 9371 | 0.108 | -0.0456 | No |
| 13 | PIK3CG | na | 9794 | 0.100 | -0.0498 | No |
| 14 | PLA2G12B | na | 10192 | 0.093 | -0.0539 | No |
| 15 | AKT2 | na | 10210 | 0.092 | -0.0470 | No |
| 16 | MAPK11 | na | 10606 | 0.085 | -0.0517 | No |
| 17 | MAPK13 | na | 10951 | 0.079 | -0.0553 | No |
| 18 | PDPK1 | na | 11113 | 0.076 | -0.0539 | No |
| 19 | PIK3CA | na | 11255 | 0.074 | -0.0520 | No |
| 20 | PLA2G2D | na | 11306 | 0.073 | -0.0476 | No |
| 21 | VAV2 | na | 11454 | 0.071 | -0.0462 | No |
| 22 | VAV3 | na | 11728 | 0.067 | -0.0487 | No |
| 23 | MAPK14 | na | 11965 | 0.064 | -0.0504 | No |
| 24 | MAPK10 | na | 12440 | 0.057 | -0.0595 | No |
| 25 | PIK3R2 | na | 12535 | 0.056 | -0.0577 | No |
| 26 | SOS2 | na | 12783 | 0.053 | -0.0606 | No |
| 27 | PLCG1 | na | 12816 | 0.052 | -0.0574 | No |
| 28 | PLCG2 | na | 12987 | 0.050 | -0.0583 | No |
| 29 | MAP2K4 | na | 13264 | 0.047 | -0.0625 | No |
| 30 | MAP2K2 | na | 14012 | 0.038 | -0.0810 | No |
| 31 | PLA2G12A | na | 14161 | 0.036 | -0.0824 | No |
| 32 | PRKCA | na | 14202 | 0.036 | -0.0807 | No |
| 33 | MAPK3 | na | 14382 | 0.034 | -0.0832 | No |
| 34 | PLA2G2C | na | 14507 | 0.032 | -0.0842 | No |
| 35 | PLA2G10 | na | 15631 | 0.022 | -0.1149 | No |
| 36 | BTK | na | 17142 | 0.007 | -0.1579 | No |
| 37 | PIK3CB | na | 17199 | 0.007 | -0.1589 | No |
| 38 | RAC2 | na | 17259 | 0.006 | -0.1601 | No |
| 39 | PLA2G6 | na | 17909 | 0.000 | -0.1788 | No |
| 40 | AKT1 | na | 18101 | -0.001 | -0.1842 | No |
| 41 | PLA2G2F | na | 18318 | -0.004 | -0.1902 | No |
| 42 | SYK | na | 18414 | -0.004 | -0.1926 | No |
| 43 | MAPK8 | na | 18612 | -0.006 | -0.1977 | No |
| 44 | PLA2G2E | na | 18859 | -0.009 | -0.2041 | No |
| 45 | MAP2K7 | na | 19405 | -0.015 | -0.2187 | No |
| 46 | MAPK1 | na | 20406 | -0.027 | -0.2454 | No |
| 47 | PIK3CD | na | 20433 | -0.027 | -0.2440 | No |
| 48 | PIK3R3 | na | 20464 | -0.027 | -0.2427 | No |
| 49 | PRKCD | na | 20569 | -0.028 | -0.2435 | No |
| 50 | JMJD7-PLA2G4B | na | 20701 | -0.030 | -0.2449 | No |
| 51 | RAF1 | na | 21720 | -0.042 | -0.2709 | No |
| 52 | PLA2G4A | na | 21803 | -0.043 | -0.2698 | No |
| 53 | HRAS | na | 21856 | -0.044 | -0.2678 | No |
| 54 | RAC1 | na | 21936 | -0.045 | -0.2665 | No |
| 55 | FCER1G | na | 22701 | -0.055 | -0.2841 | No |
| 56 | MAP2K3 | na | 23613 | -0.069 | -0.3049 | No |
| 57 | GRB2 | na | 24215 | -0.079 | -0.3159 | No |
| 58 | LAT | na | 24444 | -0.083 | -0.3158 | No |
| 59 | PRKCB | na | 25770 | -0.108 | -0.3454 | No |
| 60 | IL5 | na | 26211 | -0.117 | -0.3487 | No |
| 61 | VAV1 | na | 28682 | -0.181 | -0.4056 | No |
| 62 | LYN | na | 28768 | -0.184 | -0.3934 | No |
| 63 | NRAS | na | 30019 | -0.224 | -0.4116 | Yes |
| 64 | MAP2K1 | na | 30150 | -0.229 | -0.3970 | Yes |
| 65 | MAP2K6 | na | 30203 | -0.231 | -0.3801 | Yes |
| 66 | PLA2G5 | na | 30433 | -0.240 | -0.3676 | Yes |
| 67 | MS4A2 | na | 31419 | -0.287 | -0.3731 | Yes |
| 68 | GAB2 | na | 31969 | -0.316 | -0.3637 | Yes |
| 69 | LCP2 | na | 32895 | -0.380 | -0.3601 | Yes |
| 70 | PLA2G2A | na | 33011 | -0.388 | -0.3324 | Yes |
| 71 | IL4 | na | 33045 | -0.391 | -0.3021 | Yes |
| 72 | PRKCE | na | 33586 | -0.454 | -0.2815 | Yes |
| 73 | FYN | na | 33971 | -0.517 | -0.2513 | Yes |
| 74 | FCER1A | na | 34011 | -0.524 | -0.2106 | Yes |
| 75 | TNF | na | 34328 | -0.609 | -0.1711 | Yes |
| 76 | CSF2 | na | 34421 | -0.642 | -0.1225 | Yes |
| 77 | PLA2G1B | na | 34558 | -0.712 | -0.0695 | Yes |
| 78 | AKT3 | na | 34696 | -0.932 | 0.0009 | Yes |
Table: GSEA details [plain text format]

  

Fig 2: KEGG\_FC\_EPSILON\_RI\_SIGNALING\_PATHWAY      
 Blue-Pink O' Gram in the Space of the Analyzed GeneSet

  

Fig 3: KEGG\_FC\_EPSILON\_RI\_SIGNALING\_PATHWAY: Random ES distribution      
 Gene set null distribution of ES for **KEGG\_FC\_EPSILON\_RI\_SIGNALING\_PATHWAY**
